# A Qualitative Study Exploring Community Pharmacists' Experiences and Views About Weight Management Interventions and Services in Klang Valley, Malaysia

**DOI:** 10.3389/fpubh.2021.720928

**Published:** 2021-09-01

**Authors:** Rohit Kumar Verma, Wei Wen Chong, Nur Akmar Taha, Thomas Paraidathathu

**Affiliations:** ^1^Department of Pharmacy Practice, School of Pharmacy, International Medical University, Kuala Lumpur, Malaysia; ^2^Centre of Quality Management of Medicines, Faculty of Pharmacy, Universiti Kebangsaan Malaysia, Kuala Lumpur, Malaysia; ^3^Faculty of Pharmacy, Cyberjaya University College of Medical Sciences, Cyberjaya, Malaysia; ^4^Faculty of Health and Medical Sciences, Taylor's University, Subang Jaya, Malaysia

**Keywords:** weight management, community pharmacist, community pharmacy, obesity, overweight, intervention and views, qualitative exploration

## Abstract

**Objective:** To qualitatively explore the perspectives of community pharmacists in Malaysia on their roles in weight management, and the barriers and facilitators in the expansion of these roles.

**Methods:** A purposive sampling method was used to recruit community pharmacists in Klang Valley, Malaysia. Semi-structured individual interviews were conducted with community pharmacists, with an interview guide, from May 2018 to January 2019. The interviews were conducted in person (face-to-face). All interviews were audio-recorded with consent and transcribed verbatim. The interview transcripts were analyzed thematically, whereby emerging themes were coded and grouped into categories.

**Results:** Twenty-four community pharmacists were recruited, with years of experience in pharmacy practice ranging from 2 to 40 years. Participants described their perceptions on the different weight management interventions where they emphasized the importance of a comprehensive lifestyle intervention and viewed that it should be the first-line intervention. Participants regarded their weight management service as easily accessible or approachable since community pharmacies are often the first point of call for patients seeking advice for their conditions before consulting doctors. Barriers identified by community pharmacists were mainly organizational in nature, which included lack of private consultation rooms, lack of time, and lack of qualified staff. Participants also described the need for training in weight management.

**Conclusion:** Community pharmacists in Malaysia believed that they can positively contribute to the area of weight management. They cited multiple roles that they could play in weight management interventions and services. The roles cited include provision of education and advice, including on lifestyle modifications, drug therapy, weight loss products and supplements, and monitoring and providing referrals to other healthcare professionals. However, barriers would need to be addressed, including through pharmacist training, to strengthen and improve community pharmacists' roles and contributions in weight management service.

## Introduction

Obesity is globally recognized as a public health concern. Obesity is defined as excessive fat accumulation to the extent that it presents a risk to health. Global age-standardized estimates from World Health Organization show that in 2016, over 650 million adults (aged 18 years and older) were obese, which corresponds to about 13% of the world's adult population (1). Worldwide obesity among adults has nearly tripled since 1975, increasing from a prevalence of 4.7% in 1975 to a prevalence of 13.1% in 2016 (1). In Malaysia, a sharp increment in the prevalence of obesity over the last four decades has been documented. In 1975, about 1.4% of the Malaysian adult population was obese, but the figure has since risen to 15.6% in 2016, with more than 10 times increase in the prevalence (1). In this regard, Budgujar et al. (2) highlighted that complications associated with obesity were known to obese Malaysians; however, awareness programs are still needed to control the intensity of obesity in Malaysia.

There has been a transformation in the professional skills of the pharmacy profession over the last decades. Pharmacists, especially in the community settings, have been acknowledged for their capability to extend their role in assisting public health activities such as weight management, in addition to the traditional role of promoting quality use of medicines. Like other parts of the world, Malaysian community pharmacists are one of the most accessible health care professionals, and hence should be ideally placed to be professional advocates for public health on the front line of health care, which includes weight management.

Previous empirical research has been conducted in Australia and New Zealand to investigate the role of community pharmacists in weight management. Community pharmacists generally expressed positive views on their role in weight management. They believed that as trained health care professionals, they held a unique position compared to other community-based healthcare providers and therefore had a definite role to play in weight management (3, 4). They viewed themselves to be approachable by virtue of their ability to have regular contact with patients due to prescription dispensing, and patients are more comfortable talking to pharmacists than to general practitioners (4, 5). This feature facilitates their active role in the delivery of weight management services since they considered that such services build on the existing trust and good rapport between patients and pharmacists (4, 5).

According to previous surveys conducted, about 75–90% of the community pharmacists in Malaysia participated in the delivery of weight management services (WMS) (6, 7). Most of the community pharmacists in Malaysia in a previous study (8) reported providing extended services related to weight management, including anthropometric and physiological measurements. Such findings are encouraging, as they indicate that community pharmacists in Malaysia may have a positive perception of their roles in weight management, and thus represents a potential opportunity to further explore their role in WMS. Although a previous qualitative study has explored the provision of professional pharmacy services among community pharmacists in Sarawak (9), no research to date has qualitatively explored the views and perspectives of the community pharmacists in Malaysia regarding their role in weight management specifically.

Therefore, this qualitative study aims to explore in-depth the experiences and views of community pharmacists in Malaysia on weight management interventions and WMS. This study also aims to qualitatively explore the perspectives of community pharmacists in Malaysia on their roles in weight management, and the barriers and facilitators in the expansion of these roles.

## Methods

### Study Design

A qualitative approach was utilized to explore community pharmacists' views on weight management and their roles or involvement in weight management interventions and WMS. Data were collected *via* face-to-face semi-structured interviews, as they provided participants with an opportunity to develop and express their views. This study utilized a phenomenological approach, which is a philosophical approach that seeks to gain a description of the phenomenon from the participants' point of view (10).

### Sampling Process

A purposive sampling method was used to identify community pharmacists with different demographic variables, including the type and location of community pharmacies in which they worked. Community pharmacists who owned and/or worked in community pharmacies in the Klang Valley, Malaysia, had a minimum of 2 years' experience in the community pharmacy setting, and had previous experience in the management of overweight and obese patients, were deemed eligible. Those who were not willing to sign the consent form, had <2 years of working experience and did not provide WMS from their pharmacy were excluded. Community pharmacists who had previously registered for a weight management training program conducted by the Malaysian Pharmacists Society (MPS) were contacted and asked about their interest in participating in the study. A participant information sheet was provided for further details. Community pharmacists who expressed their interest in participating in the interview were then contacted over the phone to schedule an appointment for the interview.

The sampling process stopped when thematic saturation was reached and no additional data was obtained from the last six participants.

### Interview Guide

The interview guide was adapted from a similar study that was conducted among community pharmacists in New Zealand (4). Permission to adapt the interview guide for the study was requested from and granted by the authors. The original interview guide was modified according to the Malaysian context, in which a few questions on demographic characteristics, use of clinical practice guidelines in weight management, and requirement of training programs were either added or modified. The interview guide contained open-ended questions on perspectives of obesity, weight management interventions in community pharmacy, use of clinical practice guidelines in weight management, barriers and facilitators to expanding the role of CPs in weight management, and training needs in the area of weight management. Prior to the use in the field, the interview guide was further assessed for validity by three academic experts with experience in qualitative research and the area of community pharmacy. The interview guide was further piloted on three community pharmacists. No major changes were made after the pilot study, apart from items on demographic characteristics where the option “manager” was added under the employment section.

### Interview Process

Semi-structured interviews with the community pharmacists were conducted in person (face-to-face) after individual appointments. Prior to the interviews, the interviewer explained the study objectives and obtained written informed consent from the participants. Participants were assured of the confidentiality of their responses and their right to withdraw from the study. The interviews were conducted between May 2018 to January 2019 at the participants' workplace, specifically in the consultation room of their community pharmacies, for confidentiality purposes and to avoid any distractions. The interviews were conducted in English and were audio-recorded with additional field notes taken during the interviews. The interviews averaged 35 min in length (range: 27–42 min).

### Data Analysis

All interviews were audio-recorded with consent and transcribed verbatim. The researcher (RKV) read each transcript repeatedly while listening to the recorded data and made notations directly onto the transcripts. The interview transcripts were analyzed thematically, whereby emerging themes were coded and grouped into categories. The other researchers (CWW, NAT, and TP) verified the emerging themes and contents. The findings from the study are presented thematically.

## Results

Twenty-four community pharmacists were recruited, with 15 female pharmacists and nine male pharmacists. The age of participants ranged from 28 years to over 50 years. Their length of time in pharmacy practice ranged from 2 to 40 years. There were nine participants who worked in independent pharmacies and 15 participants who worked in chain pharmacies, respectively (Table 1).

**Table 1:** Sociodemographic and practice characteristics of participants (*n* = 24).

**Gender distribution**
***Gender frequency (n)***	
Male	9
Female	15
**Age distribution**	
***Age range frequency***	
20–29	6
30–39	9
40–49	4
50–59	5
**Highest qualification**	
Bachelor of Pharmacy	20
Master of Pharmacy	4
**Years of experience in community pharmacy**	
***Year range frequency***	
2–5 years	8
6–10 years	5
11–15 years	2
16–20 years	4
21–25 years	3
25 years and above	2
**Type of pharmacies**	
***Type frequency***	
Chain pharmacy	15
Independent pharmacy	9
**Employment status**	
***Type frequency***	
Full time employees (includes 7 managers and 1 owner)	21
Part time employees	3

Five key themes emerged from the qualitative analysis of the data. These included:

1. Identification of overweight and obese clients in community pharmacy.
2. Weight management interventions in community pharmacy.
3. Perceived roles of community pharmacists in weight management.
4. Barriers to community pharmacists' involvement in weight management.
5. Training needs in weight management for community pharmacists.

Descriptions of the key themes identified, supported by illustrative quotes, are as follows.

### Theme 1: Identification of Overweight and Obese Clients in Community Pharmacy

All participants considered body mass index (BMI) an acceptable measure to classify their clients as overweight or obese. Nevertheless, some participants would judge their clients based on physical appearance before calculating BMI. Some participants believed that BMI alone was inaccurate, and thus would also consider body composition, i.e., amount of muscle and fat in the body. Participants estimated that the proportion of overweight or obese clients encountered in their community pharmacy, as judged based on physical appearance, ranged from 10% to over 50%. Most participants believed overweight and obesity were topics that require sensitivity in how they were introduced and addressed to their clients.

Box 1 provides a selection of participant quotations related to this theme.

Box 1 **Selected quotes related to the identification of overweight and obese clients in community pharmacy.**

**Defining overweight and obesity**

“The most common one would be using BMI, with two things into consideration: weight and height of the person” [Interview 7].

“At first, I will actually look at the shape of the body. After that, I will calculate the BMI” [Interview 5].

“…I would not define my customers as obese or overweight just based on BMI alone. I will consider also the proportion of muscle and fats in the body” [Interview 7].

**Weight as a sensitive topic**

“Weight is a sensitive issue, and a lot of times we do not initiate the topic because we do not know how comfortable they are with discussing the topic” [Interview 8].

“…Not everyone is open to discuss this issue aloud. Usually we will wait for them to ask, then we intervene” [Interview 9].

### Theme 2: Weight Management Interventions in Community Pharmacy

Participants described their perceptions on the different weight management interventions in the context of community pharmacy setting. Only a few participants were aware and had read the Clinical Practice Guidelines on Management of Obesity by the Ministry of Health Malaysia (12), although none depended on the guidelines for their weight management practices. Overall, participants viewed that overweight or obese clients would benefit from a comprehensive lifestyle intervention comprising a combination of dietary and physical activity modification. Participants emphasized the importance of a comprehensive lifestyle intervention and viewed that it should be the first-line intervention.

Participants were well aware of the legal restrictions on the dispensing of drug therapy for weight management, in which they could only recommend non-prescription drugs such as orlistat as part of their weight management interventions, while a doctor's prescription is required before they could dispense prescription drugs such as phentermine. Drug therapy was mostly perceived as a last resort for participants if other weight management interventions have failed, and participants would usually refer their clients to obtain a doctor's advice before initiation of drug therapy for weight management. A few participants were mindful of the side effects of orlistat and the associated patient counseling tips to address these side effects.

All participants were familiar with the commercial weight loss products and supplements commonly sold in the pharmacy. Almost all of them would recommend these products to their clients, as they believed that the weight loss products and supplements could help their clients to achieve weight loss. Nevertheless, some participants raised potential issues surrounding the sales of these products, including the weight loss that could be of short-term due to loss of water instead of fats, as well as habit-forming potential associated with some commercial products containing laxatives. Other related problems such as overclaims of product effectiveness were also cited.

Generally, participants believed that there was a behavioral element to weight loss, in which a change of mindset was necessary for lifestyle interventions to be successful. One participant described the application of transtheoretical model as a basis to determine the client's readiness for behavioral change. Nonetheless, only one participant formally utilized cognitive behavioral therapy as part of her weight management practices.

Box 2 provides a selection of participant quotations on this theme.

Box 2 **Selected quotes related to weight management interventions in community pharmacy.**

**Dietary and physical activity modification**

“I think it's about guiding them to a proper lifestyle. Even if you're selling the best product, it won't be working without exercise and dietary intervention” [Interview 3].

“Exercise and maintain a healthy diet, those will be the core of the weight loss intervention” [Interview 8].

“First of all, counseling on lifestyle changes, because I believe this is the one that works among all other interventions” [Interview 9].

**Drug therapy**

“We don't involve so much in drug therapy. Pharmacists cannot dispense Duromine® without a prescription. It's under Group B. What we can dispense without a prescription is orlistat, a group C drug” [Interview 4].

“…I can give them drugs; drugs will be the last option if others failed. Lifestyle changes should be the first option” [Interview 2].

“…For instance, the fat absorption blocker orlistat, I find it hard to use because of the side effects” [Interview 7].

**Weight loss products and supplements**

“Products such as slimming tea give only temporary effect, you only lose water. It's not a true reflection of weight loss in terms of losing fat mass” [Interview 22].

“A lot of slimming teas contain senna, which is habit forming. Some customers lose weight rapidly after taking slimming teas in the first two weeks, but in longer term their body weight will bounce back because they are losing water, not losing fats” [Interview 8].

**Behavioral modification**

“Their mindset or how they think is very important. You intervene through the way they think and how it affects their lifestyle” [Interview 4].

“We need to do it step by step. Using the transtheoretical model, I try to find out in what stage that this person is willing to change, to see whether he is in the preparation stage, or he is in the action stage” [Interview 7].

### Theme 3: Perceived Roles of Community Pharmacists in Weight Management

Participants regarded their WMS as easily accessible since community pharmacies are often the first point of call for patients seeking advice for their conditions before consulting doctors. Participants also expressed moderate to high confidence in providing WMS in general.

Participants were unanimous of the opinion that the provision of education and advice was a vital part of their role in weight management. Apart from clients who requested WMS themselves, participants also reported routinely addressing weight issues among clients with non-communicable diseases, especially those with cardiovascular disease, diabetes, hypertension, and osteoarthritis. While all participants would like to engage in modifying the physical activity and dietary behaviors of their overweight or obese clients, some would also create awareness among their clients regarding the health risks associated with overweight or obesity as well as the importance and benefits of losing weight. Almost all participants believed they play a part in promoting weight loss products or supplements to complement their weight management advices.

Participants also saw a monitoring role in themselves, whereby some would go a step further to follow up with their clients' progress to check if they have implemented the suggested lifestyle modification. Participants generally would like to be able to refer their clients to other health care professionals such as physicians, dieticians, nutritionists, and exercise trainers as part of the multidisciplinary approach to weight management. A few participants reported referring their clients to in-house nutritionists and dietitians to receive dietary counseling. Some participants also referred clients with co-existing diseases or class 3 obesity to the physicians.

Box 3 provides a selection of participant quotations on this theme.

Box 3 **Selected quotes related to the perceived roles of community pharmacists in weight management.**

**Accessible and approachable**

“Because we are in the primary care, we are easily accessible, so a lot of clients like to come to us first” [Interview 7].

“We are easily approachable and most probably be the first line where people will come to seek help rather than going to a doctor” [Interview 9].

**Providing education and advice**

“I think the main thing that we can do for the patients is actually providing information, especially in terms of diet and exercise” [Interview 11].

“We educate people about different options and different methods of losing weight healthily” [Interview 22].

**Raising awareness**

“…We also need to educate customers a lot about the importance of losing weight because obesity is related to a lot of chronic diseases, like diabetes, hypertension, and even Alzheimer's disease” [Interview 8].

“We can actually deliver the message: the risk of obesity and the benefit if you lose your weight” [Interview 1].

**Weight loss product or supplement selling**

“So product-wise, we recommend them something to burn fats like garcinia” [Interview 6].

“Product selling will help them, because advice alone will not be enough” [Interview 21].

**Monitoring and follow up**

“…Follow up with customers to know how much they are progressing. If you just give them advice and not following up with them, you won't know if they have changed their diet, or if they have changed their lifestyle. So, follow up is the best way to know whether the customers have successfully lost weight, or whether they find your ways are helping them” [Interview 23].

**Referral to other healthcare professionals**

“If we can have access to other healthcare professionals like dietitians or fitness coaches, it will definitely be useful. It's part of the holistic approach” [Interview 16].

“In our counseling process, we do let our nutritionists teach them how to manage their diet” [Interview 8].

“If they have other comorbidities or if they haven't been undergoing their blood test, then we will refer them to a doctor” [Interview 17].

### Theme 4: Barriers to Community Pharmacists' Involvement in Weight Management

Participants identified several barriers to the effective delivery of WMS in the community pharmacy. Almost all participants revealed difficulty to introduce the potentially sensitive issue of weight due to their fear of offending their clients. They would hence normally wait for their clients to take the initiative, or bring out the topic in the context of other health conditions, especially diabetes, hypertension, or osteoarthritis. Some participants would utilize health biomarkers such as blood pressure or blood glucose rather than weight or BMI, as a way to open the discussion about weight indirectly. Relatedly, a few participants identified a lack of consultation room in the pharmacy to discuss the topic privately as a barrier to WMS involvement.

Another barrier perceived by participants was a lack of financial reimbursement. Participants would like to be remunerated appropriately, either from the clients or the employers, for their time taken to provide WMS. Participants believed that it would be time-consuming to provide proper weight counseling and therefore would forego some sales revenues if they provided WMS. Participants also perceived that their effort would be more appreciated if a fee is incurred for their WMS.

A lack of time also posed a barrier to the administration of WMS among some participants. This was especially true for community pharmacies that are always crowded with customers or those that are operated with only one or two pharmacists. A lack of qualified staff in the pharmacy was also cited as a barrier since quality time may not be devoted to their clients, with pharmacists having many tasks to handle.

Other barriers identified by participants included a lack of training on weight management, especially training programs with content adapted in the local context, and a lack of public awareness on their ability to deliver WMS, possibly due to a lack of formal weight management programs introduced by the pharmacy.

Box 4 provides a selection of participant quotations on this theme.

Box 4 **Selected quotes related to the barriers to community pharmacists' involvement in weight management.**

**Introducing weight topic**

“…Not everyone is open to discuss this issue aloud. Usually we will wait for them to ask, then we intervene” [Interview 9].

“[If] they have other comorbidities, like diabetes, [high] blood pressure, then perhaps we will recommend weight reduction. Or else, we won't interrupt” [Interview 10].

“If the customer [is] coming [for a] blood pressure or blood sugar check, and we find the reading is high, then as part of the counseling, we tell them to exercise and lose weight. From there we can start the conversation on how they can lose weight” [Interview 15].

**Lack of private consultation room**

“Having a private counseling room is a good thing. Because it is not very good to discuss weight issues in front of everyone especially when it is so crowded in the pharmacy” [Interview 5].

**Lack of remuneration**

“When we perform counseling, we may end up not doing any sales. We're actually spending time with this patient. We need to let other customers wait for our service. So I think it'll be better with a little remuneration” [Interview 3].“Some people will be more appreciative if they actually need to pay for the service. It is something that we need to look into, and it is actually quite a good initiative. Most of the time we are not going to charge a lot, even like overseas, if they charge, it is at a minimal amount, just a little appreciation of what the pharmacists can do” [Interview 18].

**Lack of time**

“Sometimes time limitation is our challenge as well, because at one time you have to serve multiple clients. There's usually one to two pharmacists only in a retail pharmacy, so during peak hours, it might be a little bit difficult for us to sit down and talk to them privately” [Interview 6].

**Lack of qualified staff**

“…The pharmacy assistant may not be really equipped with this kind of knowledge” [Interview 3].

**Lack of training**

“I think it's good to have this kind of training workshop. Because I only read from the overseas websites. We don't really know whether it works for our community or not. The way we select our food, it's actually different from European countries” [Interview 3].

**Lack of public awareness**

“If you have something more structured like the smoking cessation program, then people will be more aware of it as well, so the proactive ones will come and seek advice, and we can monitor them in a more structured way” [Interview 21].

### Theme 5: Training Needs in Weight Management for Community Pharmacists

Participants also described the need for training in weight management. More than half of the participants had completed some previous training on how to manage overweight or obese clients. All participants who had previous training on weight management agreed that this sort of training was effective, and improved their confidence in the management of overweight or obese clients. They opined that the weight management training not only refreshed their previously acquired knowledge and skills, but also provided some new insights on weight management, for instance, on the efficacy of drug therapy for weight loss and the latest commercial weight loss products and supplements in the market. Participants who had never attended any training on weight management also considered such training to be beneficial in expanding knowledge on this topic.

In terms of training needs and preferences, participants desired to have a multi-day training program to adequately cover related aspects on weight management. They would like the training session to be conducted more than once a year to share how well they have integrated the knowledge and skills learnt during previous sessions into their daily practices. Training topics deemed necessary by the participants included cognitive behavioral therapy, exercise, and dietary counseling, particularly calorie counting, as well as communication skills to initiate potentially sensitive weight topics with clients, convince overweight or obese clients to lose weight, and build rapport with clients. Most participants would like to have experience sharing sessions or case studies as part of the weight management training, especially those that involved actual clients. In addition, some participants would like the involvement of other health care professionals in weight management training, either as participants during group discussions, or as providers of the training. A few participants would like more exposure on commercial weight loss products or supplements.

Box 5 provides a selection of participant quotations on this theme.

Box 5 **Selected quotes related to pharmacists' training needs in weight management.**

**Training duration**

“Maybe they can have [a] multi-day training program for us to cover more aspects and criteria, because the previous training was too short” [Interview 20].

**Training frequency**

“Maybe they can have more sessions instead of once a year. After the first session, we would go back and implement the knowledge and skills learnt, and we share on the next session whether they are effective” [Interview 5].

**Training topics**

“…Learn about dietary counseling, communication skills, exercise regimen, and products available” [Interview 13].

“We want to know more about how to control the diet and what is the calculation for calories intake” [Interview 23].

“…Communication skills, in terms of convincing the patients into weight management” [Interview 10].

“…Include a lot of case studies, so that we can learn how to practice” [Interview 22].

“It would probably help if actual patients can come and share their experiences, and that would further tell us the achievability of whatever regimen we have set up for the patients” [Interview 11].

“I would say if there is a nutritionist or dietitian or weight management specialist in hospital who can give us training, it would give us better insight” [Interview 17].

## Discussion

To the best of authors' knowledge, this is the first qualitative study to explore community pharmacists' views on overweight and obesity, and their potential roles in weight management in Malaysia. The findings from this study support and further explain results from the previous quantitative study that aimed to assess the attitudes, practices, and perceived barriers of Malaysian community pharmacists in the delivery of WMS. In the previous quantitative study, while many of the community pharmacists were reported to actively provide WMS, they also perceived the presence of multiple barriers (8).

Findings from the current study indicated that community pharmacists were interested to play an active role when it comes to providing WMS. The community pharmacists considered themselves to be in a favorable position to provide WMS since they believed they are accessible and approachable. Beyond lifestyle modification counseling, community pharmacists also took part in the education of their clients in creating awareness regarding the health risks associated with overweight and obesity as well as the importance of shedding extra weight. In addition, with some community pharmacists providing follow up to their clients, it indicates that they would tailor their WMS according to individual needs. These findings align with global evidence for the public health roles that community pharmacists play, where they were shown to be capable of providing both population-based and individual-level public health services such as weight management (11). Moreover, despite having trouble in raising the potentially sensitive issues of weight with their clients, community pharmacists would utilize some communication techniques to overcome the difficulty, such as raising the topic in the context of other health conditions or deranged health biomarkers. The findings reciprocated with a study in New Zealand, whereby the community pharmacists would also employ similar techniques to overcome sensitivity about raising weight topics (4). With the communication techniques they employed, it is not surprising to observe that community pharmacists would routinely address weight issues among clients with comorbidities, especially cardiovascular disease, diabetes, hypertension, and osteoarthritis.

Most community pharmacists in our study would classify their clients as being overweight or obese by calculating their BMI, which is in line with the recommendations in the Clinical Practice Guidelines on Management of Obesity by the Ministry of Health Malaysia (12). However, from an anatomical and metabolic point of view, obesity is defined as excessive accumulation of body fat, and upon these grounds, the accuracy of the BMI as a determinant of body fat mass has been frequently questioned within the literature, since it has some limitations in this regard (13, 14). Therefore, it is indeed beneficial for some community pharmacists in our study to also consider the body composition, i.e., amount of body fat and muscle of their clients in addition to BMI. Nevertheless, the Clinical Practice Guidelines on Management of Obesity by the Ministry of Health Malaysia (12) was last updated in 2004, and thus it may be not surprising that none of the community pharmacists in our study depended on the guidelines for their weight management practices. This highlighted the need to update the guidelines according to contemporary evidence to serve as a reference for community pharmacists and other health care professionals to standardize their weight management practice.

It is encouraging to observe that community pharmacists in our study placed the highest importance on comprehensive lifestyle interventions, comprising of a combination of dietary and physical activity modification, among all other weight management interventions. Beneficial effects of comprehensive lifestyle interventions have long been documented within the literature, and one such example would be the Diabetes Prevention Program, which adopted a comprehensive lifestyle intervention and reported superior outcomes in diabetes prevention with lifestyle intervention compared to pharmacologic intervention with metformin (15). While community pharmacists did acknowledge the behavioral elements of weight loss, it would be ideal if they could provide a structured behavioral program that includes cognitive behavioral therapy alongside comprehensive lifestyle interventions. Behavioral-based treatment programs improve weight loss results and are associated with improvements in obesity-associated morbidity (16).

Community pharmacists in our study would carefully screen for the suitability for the initiation of drug therapy, in which they would reserve for clients who have failed other weight management interventions, and thus was in accordance with drug manufacturers' recommendations. Nevertheless, it is noteworthy to observe that almost all community pharmacists in our study engaged in the sales of weight loss products or supplements. To date, evidence to support the efficacy and most importantly, the safety of over-the-counter weight loss products and supplements is still limited (17, 18). Moreover, within the literature, community pharmacists have been subjected to close scrutiny, where criticism from consumers emerged in the social media with regards to the perceived conflicts of interest of community pharmacists selling weight loss products to increase their net revenue (5, 19). Consumers opined that community pharmacists' advice on weight management may be biased in order to profit from selling a product (19, 20). While it is understandable that community pharmacists need to maintain a viable business, and therefore stand to gain from the sales of weight loss products or supplements, this role conflict must be addressed, and a balance achieved so that pharmacists do not allow business objectives to undermine their positive public professional image.

Baseline knowledge regarding various aspects of the evidence-based obesity management such as dietary approach, physical activity recommendation, and pharmacological therapy, as well as associated health risks, would be expected for community pharmacists providing WMS. Similar to our findings, lack of training related to obesity management was occasionally singled out as a barrier to pharmacist-led interventions within the literature (21). Community pharmacists in our study deemed training in weight management to be effective and beneficial. Perceived training needs in weight management identified by community pharmacists in our study, such as behavioral therapy, exercise, dietary counseling, and communication skills, were similar to those reported in other studies (5, 22, 23). Additionally, the desire of community pharmacists for the involvement of other health care professionals in weight management training indicated the willingness of pharmacists to understand their role and thus adopt a multidisciplinary team approach to weight management.

Barriers identified by community pharmacists in our study were mainly organizational in nature, which included lack of private consultation rooms, lack of time, and lack of qualified staff. It is good for community pharmacists to acknowledge the lack of private consultation rooms as a barrier. From patients' perspective, there is a lack of sensitivity about privacy requirements for discussions of sensitive issues such as obesity in community pharmacy (24). The issue of privacy was also raised in the findings of the previous quantitative study (8), in which it was one of the factors influencing the acceptability of community pharmacist-led WMS. In addition, the availability of a consultation room for discussion and consultation of all health issues to take place was one of the patient's considerations when choosing a pharmacy in the United Kingdom (25). The Community Pharmacy Benchmarking Guideline of Malaysia, which serves as a set of standards that are required to be complied with for the purpose of community pharmacy practice set up in Malaysia, has recommended the installation of properly designated, private, and comfortable counseling areas (26).

On the other hand, previous studies aimed to evaluate pharmacist workload in community pharmacy indicated that pharmacists spent most of their time in dispensing medications, with little time allocated for patient counseling and interaction. For example, a study aimed to quantify the proportion of time the community pharmacists spent on various work activities noted that more than half (56%) of the community pharmacists' working time was dedicated to medication dispensing responsibilities, while patient counseling activities only constituted about one-fifth (19%) of pharmacists' working time (27). Therefore, it is not surprising to observe that a lack of time posed a major barrier to the administration of WMS in community pharmacy, from the perspectives of community pharmacists in this study. It has been proposed that by increasingly involving trained pharmacy assistants in the dispensatory role, it would free up some time of community pharmacists to involve more actively in patient counseling activities. Nevertheless, it remains to be determined if such an approach would undermine patient safety and be beneficial for the implementation of WMS in the community pharmacy.

Community pharmacists also cited lack of remuneration or reimbursement as one of the barriers to the delivery of WMS in community pharmacies. Similar qualitative studies in Australia and New Zealand had also unanimously highlighted the issue of lack of remuneration for time spent to administer WMS, and the difficulties to effectively administer WMS without remuneration (4). Remuneration is associated with clients' acknowledgment of the value of WMS provided by the pharmacist. There is therefore a need to demonstrate the value of community pharmacy-based WMS and evaluate the service economically by linking the aggregated clinical outcomes to the financial resources required to achieve these outcomes to demonstrate the cost-effectiveness of the service. Demonstrating the value of WMS would be instrumental in marketing the value of the service that our community pharmacists are providing and thus attract remuneration from stakeholders.

This was an in-depth qualitative study conducted with community pharmacists in Malaysia. To our knowledge, this is the first study to explore Malaysian community pharmacists' role in weight management. However, there are limitations to our study. Our findings may be limited in that the sample was confined to community pharmacists working in the Klang Valley area of Malaysia. While we expect that the findings apply to community pharmacies elsewhere in Malaysia, we do not rule out any possibility that some highlighted issues are specific to the geographical location in which it was conducted. Another possible limitation is that participants may have given professionally desirable responses. In addition, the views of community pharmacists in this study were limited to those who had experience in weight management or those who had attended prior training programs in this area. It may be possible that pharmacists with no experience in weight management may have different views than the ones reported here.

## Conclusion

In general, community pharmacists in this study believed that they can positively contribute to the area of weight management. They cited multiple roles that they could play in weight management interventions and services, including provision of education and advice, selection of weight loss products and supplements, monitoring, and providing referrals to other healthcare professionals. However, practical barriers such as the lack of space, time, and reimbursement, were acknowledged. These barriers would need to be addressed to strengthen and improve community pharmacists' roles and contributions in WMS. This may include reviewing current training programs in weight management for pharmacists, and appropriate remuneration models for community pharmacy-based WMS.

## Data Availability Statement

The original contributions presented in the study are included in the article/supplementary material, further inquiries can be directed to the corresponding author/s.

## Ethics Statement

Ethical approval for this study was obtained from National University of Malaysia, Malaysia (UKM PPI/111/8/JEP-2018-664). The patients/participants provided their written informed consent to participate in this study.

## Author Contributions

RV and WC conceived the study. RV carried out the interviews, analyzed the data, and drafted the manuscript. WC, NT, and TP assisted with data analysis and manuscript revision. All authors have read and approved the final manuscript.

## Conflict of Interest

The authors declare that the research was conducted in the absence of any commercial or financial relationships that could be construed as a potential conflict of interest.

## Publisher's Note

All claims expressed in this article are solely those of the authors and do not necessarily represent those of their affiliated organizations, or those of the publisher, the editors and the reviewers. Any product that may be evaluated in this article, or claim that may be made by its manufacturer, is not guaranteed or endorsed by the publisher.
